# Monoclonal Gammopathies of Clinical Significance—Scleromyxedema: A Case Report and Literature Review

**DOI:** 10.1002/ccr3.70741

**Published:** 2025-08-03

**Authors:** Shanshan Liang, Jingjing Zeng, Peiying Zhong, Chengyao Jia, Li Zhang

**Affiliations:** ^1^ Department of Laboratory Medicine West China Hospital, Sichuan University Chengdu China; ^2^ Department of Laboratory Medicine The First College of Clinical Medical Science, China Three Gorges University Yichang China; ^3^ Department of Laboratory 363 Hospital Chengdu China; ^4^ Department of Hematology West China Hospital, Sichuan University Chengdu China

**Keywords:** hypothyroidism, intravenous immunoglobulin, monoclonal gammopathies of clinical significance, monoclonal immunoglobulin, scleromyxedema

## Abstract

Scleromyxedema is an uncommon, chronic connective tissue disorder with an obscure etiology. It is distinguished by fibromyxoid skin lesions and elevated serum monoclonal immunoglobulin levels. The condition's rarity limits epidemiological data, making prevalence and incidence assessment difficult. Herein, we detail a clinical case of scleromyxedema, encompassing diagnosis and treatment, to augment medical understanding of this rare entity. The aim of this study is to delve into the complexities of diagnosing scleromyxedema, encompassing its diverse clinical presentations, with the goal of refining diagnostic acumen and expediting the identification process. This, in turn, mitigates the risks associated with diagnostic delays. Moreover, the imperative of instituting pertinent therapeutic measures is highlighted as a fundamental aspect of patient management, underscoring the significance of a tailored approach to treatment. Concurrently, this endeavor demands rigorous precision from clinical laboratory staff. The accurate provision of laboratory data is essential to prevent oversights and misinterpretations in diagnosing this rare condition.

**Summary**

- We describe the diagnosis and treatment of a case of scleromyxedema with hypothyroidism and find that the clinical symptoms, laboratory findings, and biopsy findings, as well as the patient's response to scleromyxedema‐guided therapy, support the diagnosis.
- Updating the diagnostic criteria to allow for concurrent thyroid dysfunction and to recognize other changes in clinical or histologic disease presentation helps clinicians to make a prompt diagnosis and initiate treatment for scleromyxedema.

## 1 Introduction

Monoclonal gammopathies of clinical significance (MGCS) represent a cadre of pathologies defined by the pathogenic presence of monoclonal immunoglobulin M proteins within the circulatory system or urinary tract, culminating in the manifestation of organ dysfunction [1, 2]. Consensus statements have significantly deepened clinicians' understanding of nonmalignant monoclonal immunoglobulin disorders. These disorders, identifiable by clinical laboratories, are acknowledged as underlying causes of various serious diseases, highlighting the importance of accurate diagnosis and a nuanced understanding in clinical practice. Discussions on MGCS enhance clinicians' diagnostic and therapeutic skills in managing these conditions and provide essential guidance to laboratory personnel. MGCS is often associated with neurological, renal, and dermatological symptoms, requiring a careful differential diagnostic process to differentiate them from other peripheral neuropathies, skin disorders, or renal diseases. This emphasizes the necessity of a collaborative approach between clinicians and laboratory staff to ensure precise identification and effective treatment of these intricate disorders [3].

Scleromyxedema is recognized as a member of the 13 conditions delineated within the MGCS consensus. Within this classification, the pathogenic contribution of the M protein in scleromyxedema remains to be fully elucidated. This disorder is distinguished by the presence of generalized papular lesions and sclerodermatous skin manifestations, which are typically concurrent with an IgG monoclonal gammopathy, highlighting its unique position within the MGCS spectrum. Diagnostic criteria: (1) extensive papular rash and sclerotic skin; (2) mucin deposition with fibroblast proliferation and fibrosis; (3) IgG M protein; and (4) absence of thyroid pathology [2, 4]. The principal therapeutic approach for managing scleromyxedema is predicated on anti‐plasma cell therapy, which targets the underlying monoclonal gammopathy [2, 5].

Unfortunately, because of the general lack of awareness of this chronic connective tissue disease, clinicians have a limited understanding of this condition compared with conditions such as primary light‐chain amyloidosis, light‐chain deposition disease, and POEMS syndrome, as mentioned in the MGCS consensus. The aim of this article is to describe the clinical case of a patient with a final diagnosis of scleromyxedema and the course of its diagnosis and treatment. The consensus has further deepened the understanding of this kind of disease and improved the understanding and diagnostic level of clinicians for such diseases.

## 2 Case History and Examination

A 30‐year‐old man developed scattered acneiform lesions on the forehead and bilateral cheeks without identifiable triggers, accompanied by extensive hyperpigmented patches over the lower back, lower abdomen, and extremities since 2017. Subsequently, progressive skin hardening and roughness emerged in the upper limbs, chest, abdomen, and lumbosacral regions, with a taut sensation and reduced skin mobility (particularly in hyperpigmented areas). Whitening of the periarticular skin around the second, third, fourth, and fifth proximal interphalangeal joints was observed upon hand extension. No joint motion limitations were noted. The patient exhibited mild periungual pallor and preserved muscle strength (grade V in all extremities), with absence of photosensitivity, oral ulcers, or muscle tenderness. Despite treatment at a local dermatology department, his condition did not improve.

## 3 Differential Diagnosis, Investigations and Treatment

The exclusion diagnosis of scleromyxedema requires differentiation from the following conditions, with key distinguishing features outlined below: (A) Systemic sclerosis (scleroderma) (i) Cutaneous characteristics: Scleromyxedema presents with generalized papular eruptions and cutaneous sclerosis, without Raynaud's phenomenon. In contrast, systemic sclerosis is characterized by symmetrical acral skin sclerosis, Raynaud's phenomenon, and visceral involvement (e.g., lungs, kidneys, gastrointestinal tract). (ii) Histopathological differences: Scleromyxedema is marked by dermal mucin deposition and fibroblast proliferation. Systemic sclerosis demonstrates homogeneous dermal collagen thickening without significant mucin deposition. (iii) Laboratory findings: Scleromyxedema is frequently associated with IgG‐type M protein. Systemic sclerosis is often linked to anti‐Scl‐70 or anti‐centromere antibodies. (B) Primary cutaneous amyloidosis. (i) Clinical manifestations: Cutaneous amyloidosis typically presents as waxy papules or plaques that are firm on palpation, without generalized cutaneous sclerosis. (ii) Histopathological features: Congo red staining positivity (indicative of amyloid deposits), whereas scleromyxedema shows Congo red negativity. (iii) Immunophenotype: Amyloidosis is predominantly associated with lambda (λ) light chains, while scleromyxedema is linked to IgG‐type M protein. (C) POEMS syndrome. (i) Multisystem involvement: POEMS syndrome is characterized by the pentad of polyneuropathy, organomegaly, endocrinopathy, monoclonal protein (M protein), and skin changes. (ii) Cutaneous manifestations: POEMS may present with skin thickening and hyperpigmentation, but is often accompanied by angioma‐like lesions or acrocyanosis. (iii) Laboratory findings: POEMS patients exhibit markedly elevated VEGF levels (typically > 500 pg/mL), whereas scleromyxedema shows mild elevation of VEGF.

In August 2017, he was admitted to West China Hospital's rheumatology clinic, where a skin biopsy showed subepidermal elongation, increased pigment cell basal layer, and lymphocytic infiltration. Dermal collagen fibers were increased, but subcutaneous tissue appeared normal.

At another hospital, he was diagnosed with scleroderma and treated with prednisone 20 mg daily without improvement. A December 2017 biopsy revealed epidermal hyperkeratosis, dermal collagen fiber hyperplasia, and chronic inflammation around blood vessels. Congo red staining was negative.

Routine tests in December 2017 were normal except for trace urinary protein. Negative tests included CMV, EBV, COOMBS, IgG4, and Bence Jones protein. ESR was 24 mm/h, CRP was negative, and serum protein electrophoresis showed M protein. Immunofixation suggested a suspicious IgG LAM type. VEGF levels were 238.39 pg/mL.

**FIGURE 1:**
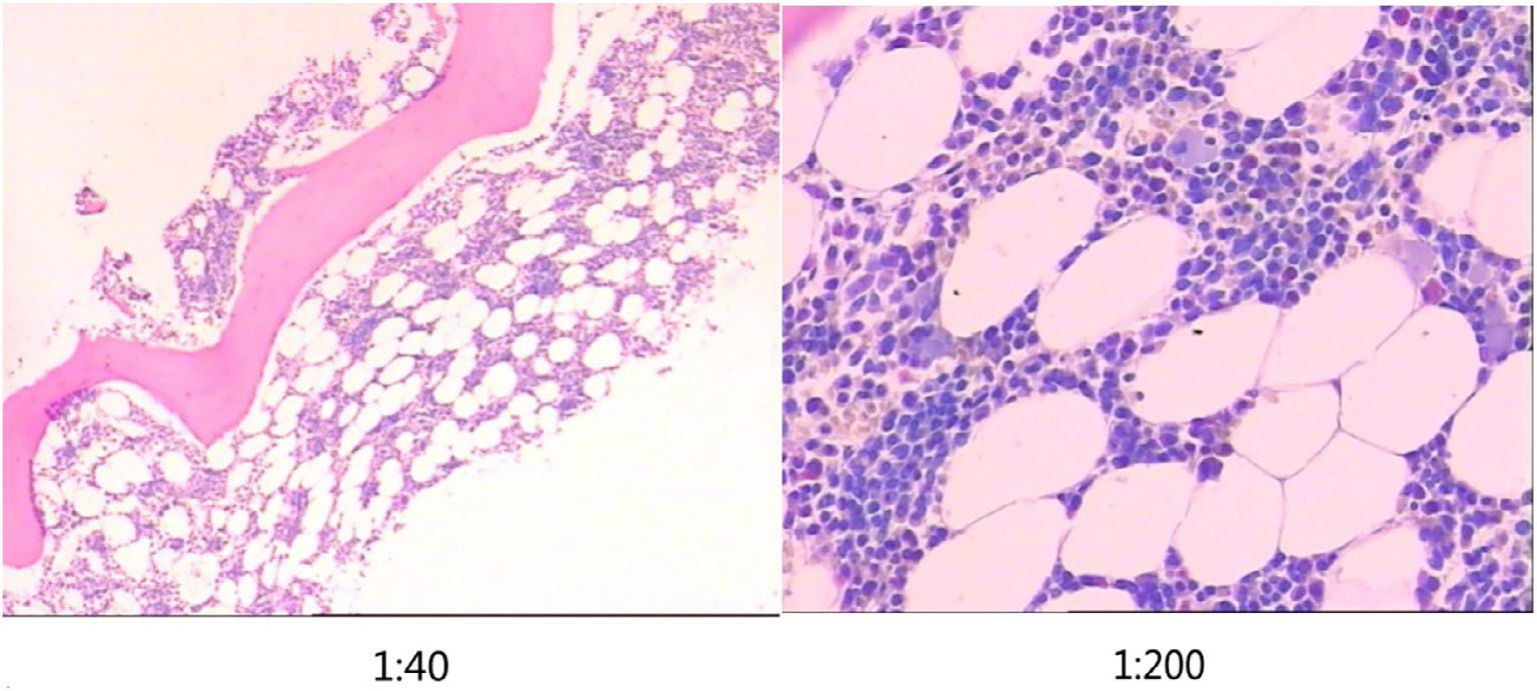
The microscopic image of bone marrow tissue biopsy. Visual observations: There was a gray brown cord‐like tissue with a length of 3.1 cm and a diameter of 0.2 cm. Histopathology: There was a small amount of cortical bone. The ratio of hematopoietic tissue to adipose tissue was about 1:1.5–2; the ratio of granulocytes was about 3:1, mainly composed of lobulated nuclei (MPO+); 5–6 megakaryocytes/HPF; there were no obvious abnormalities in cell morphology of the three lineages. In addition, a few lymphocytes and plasma cells were scattered. Special staining (FOOT staining): no increase in reticular fibers (MF‐0). Pathological diagnosis: The proliferation of bone marrow hematopoietic cells submitted for examination was low.

Doppler ultrasound indicated thickened skin with increased hardness. Electromyography showed myogenic damage in the upper limbs. Periungual microcirculation showed severe anemia and congestion without vascular inflammation. Bone marrow cytology was normal (Figure 1).

Flow cytometry (FCM) of bone marrow revealed a minor clonal plasma cell population, constituting 0.1% of nucleated cells, which expressed CD38 and CD138, with partial expression of CD19 and HLA‐DR. On January 15, 2018, the patient was admitted to West China Hospital's Hematology Department for further evaluation and management.

## 4 Conclusion and Results (Outcome and Follow‐Up)

After a comprehensive review of the patient's medical history, clinical findings, and diagnostic studies, a diagnosis of scleromyxedema was confirmed, aligning with both national and international literature. Following consultation, the patient consented to treatment with intravenous immunoglobulin (IVIG) 20 g daily for 5 days, alongside thalidomide 100 mg daily. The patient showed clinical improvement and was discharged.

Continuing the treatment at a local hospital, the patient underwent a third course of IVIG therapy from June 22, 2018, with a dosing regimen of 20 g on Days 1–2 and 10 g on Days 4–5, complemented by thalidomide 100 mg at night. Subsequent pulse therapies were initiated on September 28, 2018, followed by a fifth course in May 2019, and a sixth course in May 2020.

The patient had been managing hypothyroidism for over 7 years with Euthyrox 50 μg daily and had controlled hypertension for more than 2 years, with peak readings of 140/110 mmHg, using levamlodipine tablets (SHI HUI DA) 2.5 mg daily. Post‐sixth pulse therapy, the patient was discharged with stable vital signs and was advised to continue regular follow‐ups in the hematology clinic.

The patient, on a follow‐up visit on December 25, 2020, showed complete resolution of rash and skin softening, continuing on thalidomide 100 mg qn. In April 2021, M protein turned negative, but by September, it reappeared as a 5.6% IgG λ type. Thalidomide was continued.

In April 2022, a weak λ light chain was suspected. By December 2022, bone marrow showed 0.4% clonal plasma cells and a weakly positive 1.5% M protein, IgG λ type. With a planned pregnancy, thalidomide was suspended for 3–6 months from the patient's fertility perspective, with sperm cryopreservation as an option. In March 2023, a weakly positive 1.6% M protein and IgG λ type were noted.

## 5 Discussion

Scleromyxedema is a rare, chronic, and progressive connective tissue disorder, categorized within the spectrum of primary cutaneous mucinosis. It predominantly affects middle‐aged adults, occurring without racial or gender bias, and its exact incidence remains unknown [6]. Our case patient experienced the onset of disease at 24 years of age, which is close to the youngest reported age of onset, documented at 23 years old [7].

Skin mucin deposition, enhanced fibroblast proliferation, and significant collagen deposition characterize the histological presentation of the disease. The precise etiology remains elusive, though it is hypothesized that the disorder may arise from cytokine dysregulation, which in turn stimulates glycosaminoglycan synthesis and fibroblast activity [8].

The aforementioned four criteria—originally put out by Montgomery and Underwood in 1953 [9] and updated by Rongioletti and Rebora in 2001 [10]—are used to make the diagnosis of scleromyxedema. In 2017, Nofal et al. [11] presented updated guidelines that included changes in clinical, histological, and ancillary features of scleromyxedema, and in 2020, Hazan et al. [12] argued that diagnostic criteria should not exclude the presence of thyroid disease. The case reported by Lauren Michelle et al. [13] also had hypothyroidism, which was similar to the case reported in our report. Although thyroid disease was also excluded by the domestic 2022 consensus [2], the patient's clinical symptoms, laboratory examination results, and biopsy results all supported the diagnosis of scleromyxedema. Moreover, the patient's response to therapy for scleromyxedema, even with concurrent hypothyroidism, confirms the diagnosis in this instance. We assert that prompt recognition and diagnosis of scleromyxedema are crucial for initiating targeted therapy, which can substantially reduce the risk of severe morbidity and mortality associated with the condition.

While standardized treatment guidelines for scleromyxedema are lacking, high‐dose intravenous immunoglobulin (IVIG) is recommended as a first‐line therapy. It can be administered alone or in combination with systemic steroids and/or thalidomide [8]. Most cases of scleromyxedema have been successfully treated at home and abroad [14, 15, 16]. Young patients, in particular, have a remarkable response to IVIG administration [17]. The 30‐year‐old male patient demonstrated significant improvement in both systemic and cutaneous symptoms following IVIG treatment. He is currently undergoing thalidomide maintenance therapy and remains under continued follow‐up.

This case review and consensus learning will enhance clinicians' understanding of this rare disease. Concurrently, it imposes stricter demands on clinical laboratory personnel. Delivering accurate and reliable laboratory data is crucial to prevent missed or misdiagnoses.

In addition, we present this case to further support the recommendations of previous authors [12, 13] to update diagnostic criteria to allow for concurrent thyroid dysfunction and to identify other changes in clinical or histological disease presentation, helping clinicians to promptly diagnose and initiate scleromyxedema‐directed therapy, thereby reducing morbidity and mortality. Regarding the 2022 domestic consensus on the criteria for excluding thyroid disease in the diagnosis of scleromyxedema, we will also pay further attention to the follow‐up of our case to obtain a deeper understanding of this kind of disease.

The recognition of clinically significant MGCS has broadened our comprehension of the pathophysiology across this range of disorders. Besides the kidneys, the peripheral nervous system, skin, and eyes are the three primary organ systems impacted by MGCS. Challenges in diagnosing MGCS arise from scarce clinical data, the infrequency of certain syndromes, and a general lack of awareness among medical professionals. The 2022 consensus study aims to enhance understanding and diagnostic accuracy of these conditions, thereby reducing the incidence of missed and misdiagnoses.

The study from Theves et al. [18] highlights the efficacy of plasma cell‐directed therapies, such as lenalidomide and daratumumab, in achieving profound hematological and dermatological responses in severe or relapsed scleromyxedema. Notably, two patients treated with daratumumab combined with lenalidomide and dexamethasone demonstrated sustained remission, suggesting the potential of anti‐CD38 monoclonal antibodies in dual‐targeted regimens. Additionally, the use of bortezomib‐based regimens (e.g., VCD) in refractory cases underscores the role of proteasome inhibition, though tolerance remains a challenge. Future research should prioritize multi‐center trials to validate the durability of these therapies and optimize combination protocols. For instance, exploring daratumumab in earlier treatment lines or integrating novel immunomodulatory agents like pomalidomide may further reduce HDIVIg dependence [18]. Longitudinal studies are critical to assess the sustainability of remission after therapy withdrawal and to identify biomarkers (e.g., serum monoclonal component levels) that correlate with relapse risk. Collaborative efforts, such as international registries, could aggregate data on rare complications (e.g., dermato‐neuro syndrome, cardiac involvement) and refine consensus guidelines for monitoring and therapy escalation. Finally, molecular profiling of clonal plasma cells may elucidate disease mechanisms and guide personalized approaches, as proposed in recent studies [18].

## 6 Limitations

This study has several limitations. First, as a single‐case report, it inherently lacks the statistical power to generalize findings to broader populations. Second, genetic analyses were not performed, which could have provided deeper insights into potential predisposing factors or molecular mechanisms underlying scleromyxedema. Third, long‐term disease management remains challenging due to the relapsing–remitting nature of the condition and the need for sustained immunosuppressive therapy, which carries risks of adverse effects. Additionally, while intravenous immunoglobulin (IVIG) and thalidomide demonstrated efficacy in this case, the absence of standardized treatment protocols complicates the optimization of therapeutic strategies. Future multi‐center studies with larger cohorts are warranted to address these gaps.

## Author Contributions

**Shanshan Liang:** writing – original draft, writing – review and editing. **Jingjing Zeng:** writing – original draft, writing – review and editing. **Peiying Zhong:** writing – original draft, writing – review and editing. **Chengyao Jia:** writing – original draft, writing – review and editing. **Li Zhang:** writing – original draft, writing – review and editing.

## Ethics Statement

The study was approved by the Institutional Ethics Committee of West China Hospital of Sichuan University and complied with the Declaration of Helsinki. Written informed consent was obtained from the subject. Informed consent for publication of the clinical details was obtained from the patient.

## Conflicts of Interest

The authors declare no conflicts of interest.

## Data Availability

All data generated or analyzed during this study are included in this article.
